# Amyotrophic Lateral Sclerosis (ALS) prediction model derived from plasma and CSF biomarkers

**DOI:** 10.1371/journal.pone.0247025

**Published:** 2021-02-19

**Authors:** Radhika Khosla, Manjari Rain, Suresh Sharma, Akshay Anand

**Affiliations:** 1 Department of Neurology, Neuroscience Research Lab, Post Graduate Institute of Medical Education and Research, Chandigarh, India; 2 Department of Statistics, Panjab University, Chandigarh, India; First Affiliated Hospital of Dalian Medical University, CHINA

## Abstract

Amyotrophic Lateral Sclerosis (ALS) is a degenerative disorder of motor neurons which leads to complete loss of movement in patients. The only FDA approved drug Riluzole provides only symptomatic relief to patients. Early Diagnosis of the disease warrants the importance of diagnostic and prognostic models for predicting disease and disease progression respectively. In the present study we represent the predictive statistical model for ALS using plasma and CSF biomarkers. Forward stepwise (Binary likelihood) Logistic regression model is developed for prediction of ALS. The model has been shown to have excellent validity (94%) with good sensitivity (98%) and specificity (93%). The area under the ROC curve is 99.3%. Along with age and BMI, VEGF (Vascular Endothelial Growth Factor), VEGFR2 (Vascular Endothelial Growth Factor Receptor 2) and TDP43 (TAR DNA Binding Protein 43) in CSF and VEGFR2 and OPTN (Optineurin) in plasma are good predictors of ALS.

## Introduction

Amyotrophic Lateral Sclerosis (ALS), a multi-system neurodegenerative disorder, is a rare motor neuron disease. The symptoms involve the degeneration of upper and lower motor neurons along with weak muscular strength, lost ability of movement and speech leading to total or partial paralysis. Talbott et al have reported the global prevalence of disease to be 6/100,000 individuals [1] with an approximate male: female ratio of disease incidence to be 1:3 [2]. Riluzole is the only known Food and Drug Administration (FDA) approved drug for ALS which gives only symptomatic relief to patients [3].

Diagnosis and prognosis of ALS is dependent upon clinical investigations. Various models have been proposed to predict the survival and prognosis of the disease [4–8]. These can also help in analysing the course of disease progression during clinical trials. Diagnosing ALS using clinical investigations can take a long time that leads to certain delay in starting the treatment of patients. Hence, diagnosing ALS at the earlier stages of the disease is immensely important.

Biomarkers are the measures that can provide significant information about the disease prediction or progression. In our previous study, out of a panel of six biomolecules including Vascular Endothelial Growth Factor (VEGF), VEGF receptor 2 (VEGFR2), Angiogenin (ANG), Optineurin (OPTN), Transactive response DNA binding protein 43 (TDP43) and Chemokine Ligand 2 (CCL2), five biomolecules were found to be significantly altered in plasma [9]. In another study, Cerebrospinal Fluid (CSF) from the same cohort (approx. half of the patients) was analysed for the same six molecules [10]. Three of the molecules, involved in the angiogenic and neuroprotective pathway, were found to be significantly altered. In the absence of a single biomarker for the disease diagnosis, analysing a panel of molecules in various biofluids simultaneously can have predictive value for ALS. The previous logistic regression model was proposed based on VEGF and CCL2 mRNA levels, serum levels of CCL2 and consumption of smoking and alcohol data with high sensitivity and specificity [11]. However, the model included fewer numbers of patients and only three biomolecules i.e VEGF, CCL2 and lipid hydroperoxides were studied.

We aimed to develop a predictive statistical model based on new panel of six bio-molecules analysed in Plasma and CSF of patients along with their socio-demographic characteristics of patient population. The forward stepwise (binary likelihood) logistic regression model proposed in the present study can predict ALS with high sensitivity and specificity.

## Methods

### Participants

Total 239 participants (107 ALS and132 controls) were recruited. All the participants provided informed consents. The study approval was provided by the Institutional ethical committee (IEC approval number PGI/IEC/2014/2249) of the Post Graduate Institute of Medical Education and Research (PGIMER), Chandigarh, India. ALS patients were recruited from the Neurology outpatient Department. Among all participants, biomarkers were estimated in 187 unhaemolysed plasma and 86 CSF samples. Socio-demographic data was also collected for variables such as gender, age, BMI, smoking, alcohol, diet, ALSFRS-R, disease onset and duration of the disease. The criterion for including samples in statistical analysis was that there is no missing value for any of the 21 variables. The logistic regression for developing the model was developed considering 23 ALS patients and 14 controls. The patients were diagnosed clinically and recruited on the basis of revised El Escorial criteria [11–13]. All the patients were found to be sporadic on the basis of family history. According to el Escorial criteria, the patients were categorised as definite/possible/probable ALS.

### Statistical analysis and modelling

All the statistical tests were done using Statistical Product and service Solutions (SPSS v 23.0 SPSS Inc., Chicago, USA). Descriptive statistics was applied to analyse the distribution of data for various parameters. Binary Logistic regression model was applied for predicting risk of ALS based on the quantitative and qualitative data collected. Total 21 variables were tested including the proteins levels such as VEGF, VEGFR2, ANG, OPTN, TDP43 and CCL2 in plasma and CSF and socio-demographic details such as gender, age, BMI, smoking, alcohol, diet, ALSFRS-R, disease onset and duration of the disease. A forward stepwise (likelihood ratio) method was used for applying the model.

## Results

### Development of ALS predicting logistic regression model

Forward stepwise (likelihood ratio) binary logistic regression analysis was performed to compute the predicted risk (P) of ALS with the help of the following equation

$$P=\frac{1}{1+e^{-Y}}$$

Where, Y is model score.

Before calculating Y, Hosmer–Lemeshow goodness of fit statistic was applied to test whether the given data fits to the logistic model. Null hypothesis (H_0_) indicating that the given data fits well to the logistic model was tested and chi square (χ^2^) = 0.468, degree of freedom (df) = 8 and p = 0.994 suggests that the logistic model is adequately in agreement with the null hypothesis and fits the data.

Omnibus test of model coefficients also confirmed that forward stepwise (likelihood) binary logistic regression is highly appropriate analysis for generating predictive equation. Omnibus test yielded χ^2^ = 255.58, df = 7 and p<0.001.

The Wald test showed that out of the 21 predictors only 7 predictors were significant and can predict the risk of ALS. Following equation was obtained from the Beta values obtained by Wald test and are presented in Table 1.

**Table 1:** Significant independent variables revealed by maximum likelihood method for logistic regression equation.

Variables	Beta (β)	Standard error	Wald	Degree of freedom	p-value
Age	0.151	0.040	14.601	1	<0.001
BMI	-0.243	0.106	5.296	1	0.021
VEGFR2 plasma level	-2501.477	668.648	13.996	1	<0.001
OPTN plasma level	93.109	33.889	7.548	1	0.006
VEGF CSF level	-0.244	0.071	11.777	1	0.001
VEGFR2 CSF level	3.184	0.639	24.841	1	<0.001
TDP43 CSF level	-0.130	0.038	11.378	1	0.001
Constant	-57.040	13.953	16.713	1	<0.001

Abbreviations: BMI Body Mass Index, VEGFR2 Vascular Endothelial Growth Factor Receptor 2, OPTN Optineurin, VEGF Vascular Endothelial Growth Factor, CSF Cerebrospinal Fluid, TDP 43 Transactive Response DNA Binding Protein 43.

Model Score (Y) = -57.04 + 0.151 Age—0.243 BMI -2501.477 VEGFR2 plasma level + 93.109 OPTN plasma level—0.244 VEGF CSF level + 3.184 VEGFR2 CSF level—0.130 TDP43 CSF level.

Adequacy of the logistic regression model was supported by -2 log-likelihood method with χ^2^ = 51.73. Coefficient of determination (R^2^) was computed using Cox and Snell’s, and Nagelkerke’s R^2^, to check the association of variables in current logistic regression model. R^2^ close to 1 suggests strong association of selected independent variables with dependent variables. The present logistic regression model has Cox and Snell’s R^2^ = 0.684 and Nagelkerke’s R^2^ = 0.912.

### Validity of logistic regression model

**Fig 1:**
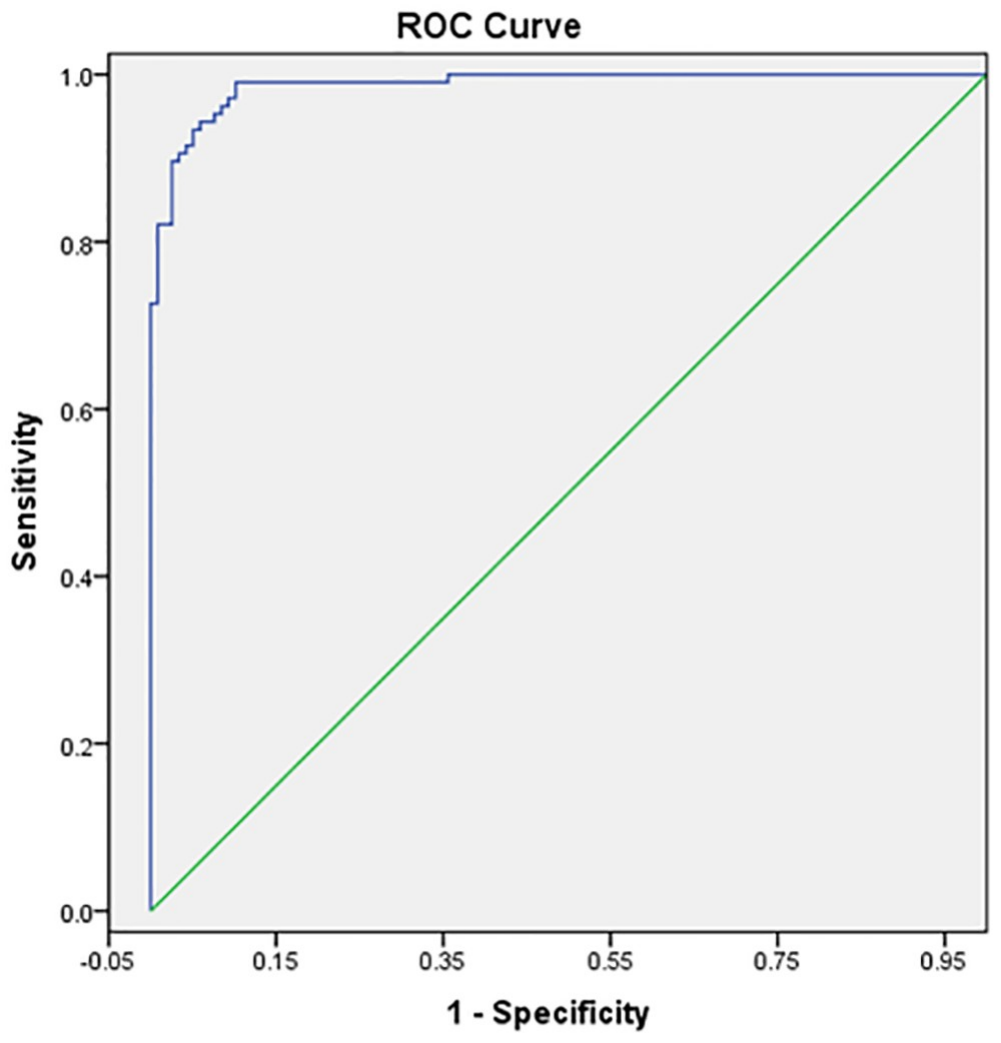
Receiver operating characteristic (ROC) for the forward stepwise binary logistic regression model for predicting ALS risk.

The Correct classification using logistic regression model of ALS was 94%. Sensitivity and specificity of the logistic regression model was 98% and 93%, respectively. Receiver operating characteristic (ROC) curve with 7 predictive variables revealed that the model for predicting ALS risk is an excellent model, as the area under the curve was 99.3% (Fig 1). As expected the ROC curve has low standard error of 0.003 with 95% confidence interval as 0.986–0.999 (Table 2).

**Table 2:** Validity of logistic regression model.

Area Under the Curve				
Test Result Variable(s): Predicted probability				
Area	Standard Error^a^	p value^b^	Asymptotic 95% Confidence Interval	
			Lower Bound	Upper Bound
0.993	0.003	<0.001	0.986	0.999

^a^. Under the nonparametric assumption

^b^. Null hypothesis: true area = 0.5

## Discussion

ALS is a motor neuron disease caused by degenerative changes in the motor neurons of spinal cord and cortical regions in brain. The degenerated neurons lead to impaired synaptic connections with muscles leading to paralysis in patients. In severe cases this may lead to respiratory failure causing fatality [14]. 10% of the cases have family history of ALS and are known as familial ALS cases. However, 90% of the cases are sporadic and occur because of mutations in varied number of genes. Most commonly associated cases are of C9ORF72 and SOD1 genes [15]. The variability in the pathophysiology of disease makes it a multi system degenerative disease or a multivariate disease. This multi system degeneration obscures the diagnosis, prognosis and treatment strategies even more. However, early prediction of the disease and predictive prognostic patterns for individualised or cohorts of patients can help in finding effective treatment strategies for ALS and delaying ALS-related adversity and mortality.

Failing to find a single molecule or factor for diagnosis, opting for a panel of markers or some statistical models or equations can help in prediction of ALS. Such models can also help in analysing the prognosis of disease in patients. We have analysed such a panel of markers that are involved in pathways of pathology of disease. VEGF [16–18], VEGFR2 [19, 20] and ANG [21, 22] have been studied in respect to angiogenic pathways as well as in neuroprotective pathways. The dysregulated levels of angiogenic molecules can cause oxidative stress. Oxidative stress has been linked to neuronal degeneration in various studies [23, 24]. The soluble counterpart of another VEGF receptor (sVEGFR1) has been associated with ALS in previous lab studies. These molecules have been shown to be neuroprotective in various studies. Other two molecules OPTN [25] and TDP43 [26] are associated with proteinopathy, which is a characteristic of ALS. Both molecules have been found to be accumulated in protein inclusions in the cytoplasm of neurons. Also TDP43 levels have been measured in CSF and serum of ALS patients as it is a major content of motor neuron inclusions. CCL2 is the main molecule of neuroinflammation pathway, which is also a characteristic feature of ALS [18, 27–29]. VEGF and CCL2 have also been shown to contribute significantly to the regression model developed by Gupta et al [11]. In this study, the molecules have been studied in plasma and CSF, both. Since there are theories that CSF is the fluid that may carry the pathogenic markers responsible for degeneration of motor neurons, measurement of proteins in CSF is important.

In combination with molecular markers simple socio-demographic factors such as age and BMI can also contribute significantly as predicting factors in logistic regression model. Also the protein levels in serum [30, 31] and SNPs [29, 32–34] can be seen in various candidate molecules and then the biomarker potential of these molecules can be explored using such regression models. Analysing the panel of markers in plasma and CSF both along with socio-demographic and clinical details can add more value to the model developed and improve the sensitivity and specificity of the model. The model has the potential of prediction of ALS even though other prognostic and survival prediction models have also been developed in the past years.

The model should be tested on larger cohorts to study the validity and predictability. Also, the markers should be analysed in CSF to blood and at cellular level (in the form of gene expression) to add to the validity of models.

## Conclusion

The proposed forward stepwise (binary likelihood) logistic regression model has shown high sensitivity and specificity. Also, the 99.3% area under the curve is indicative of the excellence of the model in predicting the risk of ALS. However, lesser sample size can be shortcoming of the predictive model. Developing such risk predicting models or combined models that can predict the risk of disease as well as survival using bigger cohorts of participants can be helpful in understanding the aetiology of ALS.

## Supporting information

S1 Data (XLSX)

## Abbreviations

ALS FRS R: Revised ALS Functional Rating Score
ALS: Amyotrophic Lateral Sclerosis
ANG: Angiogenin
BMI: Body Mass Index
CCL2: Chemokine Ligand 2
CSF: Cerebrospinal Fluid
ELISA: Enzyme Linked Immunosorbent Assay
FDA: Food and Drug Administration
OPTN: Optnieurin
PGIMER: Post Graduate Institute of Medical Education and Research
ROC: Receiver Operating Characteristics
SPSS: Statistical Product and service Solutions
TDP43: Transactive response DNA Binding Protein 43
VEGF: Vascular Endothelial Growth Factor
VEGFR2: VEGF Receptor 2
